# Developing Stress Management Programs in a Public Primary Healthcare Institution: Should We Consider Health Workers’ Sociodemographic Groups?

**DOI:** 10.3390/medicina56040162

**Published:** 2020-04-03

**Authors:** Daiva Dudutienė, Audronė Juodaitė-Račkauskienė, Rimantas Stukas

**Affiliations:** Department of Public Health, The Institute of Health Sciences, The Faculty of Medicine, Vilnius University, LT-03101 Vilnius, Lithuania

**Keywords:** occupational safety and health, job stress, psychosocial risks, organisational interventions, health professionals or health workers, wellbeing at work

## Abstract

*Background and Objectives*: An essential part of occupational stress management is identifying target groups and developing a wellbeing program that tailors interventions to the specific needs of the target groups. This study aims to explore whether psychosocial risk determinants and organizational intervention objects differ across employees’ groups based on sociodemographic factors in a Lithuanian public primary healthcare institution. *Methods*: All 690 health workers of the institution were invited to participate (response rate 68%) in a cross-sectional survey between February and March 2017. The questionnaire contained items related to sociodemographic factors (gender, age, job seniority, education, and occupation), 14 psychosocial risk determinants, and 10 organisational intervention objects. *Results*: The results of the study showed that differences by gender were not statistically significant except for one organisational intervention object (work–life balance). Only a few organisational intervention objects (justice of reward, matching to the job demand, and variety of tasks) had mean rank scores differing statistically across age and job seniority groups. Five organisational intervention objects (work–life balance, variety of tasks, communication, manager feedback, and stress management training) had mean rank scores differing statistically across education groups, and all organisational intervention objects (except stress management training) had mean rank scores differing statistically across occupational groups. Regarding psychosocial risk determinants, excessive work pace had mean rank scores differing statistically across age and job seniority groups. Four (overtime, unclear role, conflicting roles, and being under-skilled) and six psychosocial risk determinants (work overload, overtime, tight deadlines, unclear role, being under-skilled, and responsibility) had mean scores differing statistically across education and occupational groups, respectively. Statistical significance was considered with *p*-value < 0.05 and 95% confidence interval. *Conclusions:* The findings showed that different psychosocial risk determinants and organizational interventional objects were emphasized by different sociodemographic groups in the institution, but they did not impact groups in the same measure. Therefore, it is crucial to start by determining the risk group’s specific needs before developing and implementing stress management programs.

## 1. Introduction

Patients have high expectations of the speed and quality of healthcare, so demand and time pressures are increasing. The digital transformation of health services and fast changing work environment are additional challenges for health workers. Problems observed in a number of healthcare institutions include increased staff shortage, staff turnover, sickness absenteeism, high workload, and work demands. In addition, the health workforce itself is ageing and there are problems of retention due to demanding working conditions and relatively low pay in some countries. Thus, it comes as no surprise that the health workforce is an occupational group whose wellbeing and job performance are adversely affected by job stressors. Many researchers have investigated the relationship between job stressors and job performance. Many studies also showed consistently negative relationships between job stressors and job performance [1,2]. Policies that decrease job stress have the potential to improve the health of employees and to reduce the burden of absenteeism due to illness [3,4].

However, there is no simple solution to managing job stressors and to improving employees’ health and wellbeing at work. At an EU level, it is the employers’ obligation to improve the working environment by dealing with all types of risk, including psychosocial risks [5]. Over the last century, many studies have been conducted on how to better implement the welfare policy at work. Numerous qualitative and quantitative studies have explored how to improve psychosocial work environment in many countries. A systematic review of the job stress intervention evaluation literature in 1990–2005 [6] has discovered that organisationally directed interventions are more beneficial than individually directed interventions. Some studies have documented the positive effects of the organisational stress management interventions [7,8,9], while others have documented little, no, or negative effects of these interventions [10,11]. Some researchers have pointed out that variety of factors—individual personality, job profile, workplace culture, standards, and/or regulations in different areas—may lead to different outcomes [12,13]. Debate continues about the best approaches for psychosocial risk management at work. In the last century, the majority of studies aimed at finding a one-size-fits-all approach to the design of wellbeing at work [14]. There is still a gap between theoretical knowledge on psychosocial risk management and respective practical applications. Therefore, researches have recently turned to analysing preconditions and to examining the role of intervention by the organisation to the individuals within it [15,16]. Results of a meta-analysis of person–job, person–organisation, person–group, and person–supervisor fit “provide strong evidence for the importance of multiple types of fit for work-related attitudes and behaviours” [17]. A multiple approach may enable an employer to make “an assessment of its relative position (compared to “the average employee” and to specific norm-scores of the branch) and to make internal comparisons between departments or groups in the organisation (on the basis of age, gender, blue versus white collar, and so on)” [14]. This study aims to contribute to this growing area of research by exploring the importance of the sociodemographic factors in developing a psychosocial risk management strategy to improve employee wellbeing in the organisation. Thus, the study was designed to assess the differences of groups’ attitudes (on the basis of gender, age, job seniority, education, and occupational status) toward psychosocial risk determinants and organizational intervention objects in order to obtain information that is not captured by the dominant evaluation paradigm [18,19].

## 2. Materials and Methods

### 2.1. Study Sample and Design

This study is a cross-sectional study designed to examine health workers’ attitudes toward the psychosocial risk determinants and organizational intervention objects using a complex quantitative tool based on sociodemographic factors. All data was collected by paper questionnaires from February to March 2017. The sample consisted of 467 health workers employed in one of the largest primary healthcare institutions (the eight healthcare institutions were merged into one in 2002) in Lithuania. The institution employed 690 health workers in 2017. All health workers were invited to participate in the study. At the start of the study, health workers were provided with information about the study and 690 paper questionnaires were distributed; 468 questionnaires were returned compiled, one of which was damaged; 467 questionnaires (response rate 68%) were suitable for the analysis of the research results.

### 2.2. Ethical Considerations

Participation in the survey was voluntary with guaranteed anonymity and confidentiality under Lithuanian law which does not require ethical approval for this type of study. The study was authorised by the administration of the institution.

### 2.3. Instruments/Measures

The self-administrated questionnaire was used as an instrument for data collection. The instrument contained items adopted from an established questionnaire that was used for complex stress management study at Lithuanian automation and electrotechnical companies [20]. The questionnaire consisted of 67 items, divided into three parts related to psychosocial risk determinants, organisational intervention objects, and sociodemographic factors; Cronbach’s alpha was 0.89 for the general scale. However, before using the instrument in this study, it was subjected to a pilot testing where 50 questionnaires were administered to 50 health workers in the institution. After the pilot study and a discussion with the administration of the institution, an abridged and adapted to health work version of the validated instrument was used:

-Fourteen items for psychosocial risk diagnosis: hazardous working conditions; work overload; excessive work pace; overtime; tight deadlines; unclear role; conflicting roles; being under-skilled for a job; responsibility for decision making and actions; lack of control over work pace; lack of control over work method; and interpersonal relationships (harassment, conflicts, and tension). Cronbach’s alpha = 0.702;

-Twenty-seven items for organisational intervention object ascertainment: social support (5 items; Cronbach’s alpha = 0.704), organisational support (4 items; Cronbach’s alpha = 0.708), participation in decision making (3 items; Cronbach’s alpha = 0.66); communication (4 items; Cronbach’s alpha = 0.728); justice of reward (2 items; Cronbach’s alpha = 0.756); manager feedback (5 item; Cronbach’s alpha = 0.738); stress management training (1 item); work–life balance (1 item); skills/abilities matching to the job demands (1 item); and variety of tasks (1 item);

-Five groups on the basis of sociodemographic factors: gender (male, female), age (coded on four levels: ≤30, 31–40, 41–50, and >50 years), job seniority (coded on four levels: ≤3, 3.01–5, 5.01–10, and >10 working years), educational level (coded on three levels: university degree, higher school/college degree, and other degree), and occupational status (coded on four levels: heads of units, doctors, nurses, and other health workers (ergotherapists, masseurs, etc.)).

In total, the questionnaire consisted of 46 items, which were rated on a 5-point frequency scale ranging from 1 (*never or strongly disagree*) to 5 (*always or strongly agree*). Prior to analyses, some items of the questionnaire were reversed so that higher scores showed greater negative impact of psychosocial risk determinant and more relevant to organisational intervention object.

### 2.4. Statistical Analysis

Data were analyzed using the statistical software package IBM SPSS Statistics (Vilnius University, Vilnius, Lithuania). In all analyses, statistical significance was considered with *p*-value < 0.05 and 95% confidence interval (CI). A descriptive analysis was carried out to examine the sociodemographic groups of employees in the institution. Nonparametric tests for comparisons of the groups (the Mann–Whitney test for comparisons of two groups and the Kruskal–Wallis test for comparisons of more than two groups) were used then.

## 3. Results

Table 1 describes sociodemographic groups in the institution. As is the case for many Lithuanian healthcare institutions, the results showed a predominance of women (94.9%). Health workers were aged 22 to 73 years old. Almost half of health workers (47.9%) were over 50 years of age. More than half of all health workers (52.9%) had university degrees, 38.5% of health workers had higher school degrees, and 8.6% of health workers had other levels of education. The majority of health workers were nurses (43.9%), followed by doctors (28.3%), other health workers (21.6%), and heads of units (6.2%). Health workers’ seniority ranges are from a few months to 48 years; 350 of the health workers worked over 10 years (76.1%).

Table 2, Table 3, Table 4, Table 5, Table 6, Table 7, Table 8, Table 9, Table 10 and Table 11 present the attitudes of the sociodemographic groups to the psychosocial risk determinants and organizational intervention objects (mean ranks, sample sizes (N), U values (the Mann–Whitney U-test) or χ2 values, with k − 1 degrees of freedom,( the Kruskall–Wallis test) and significance levels (p)).

*Gender.* The observed differences by gender in the institution was not statistically significant except work–life balance. Women showed a higher average work–life balance score than men (Table 2; Table 3).

*Age.* Only excessive work pace as psychosocial risk determinant (Table 4) and justice of reward and matching to the job demands as organisational intervention objects (Table 5) had mean rank scores differing statistically across age groups. Excessive work pace increased consistently with age, with the oldest group of health workers (>50) scoring highest on this factor. Justice of reward decreased consistently with age, with the youngest group of health workers (≤30) scoring highest on this factor while the oldest group of health workers (>50) gave a higher score for matching to the job demands than other age groups.

*Job seniority.* Excessive work pace as psychosocial risk determinant and justice of reward, matching to the job demands, and variety of tasks as organisational intervention objects had mean rank scores differing statistically across job seniority groups. Health workers who had more than 10 years tenure gave the highest scores for excessive work pace (Table 6), and health workers who had three to five years tenure gave the highest scores for all three organisational intervention objects (Table 7).

*Education.* Four psychosocial risk determinants (overtime, unclear role, conflicting roles, and being under-skilled) and five organisational intervention objects (work–life balance, variety of tasks, communication, manager feedback, and stress management training) had mean rank scores differing statistically across education groups. Health workers who hold university degrees gave the highest scores for overtime, health workers who hold higher school degrees gave the highest scores for being under-skilled, health workers with other levels of education gave the highest scores for unclear role and conflicting roles (Table 8). Mean rank results also showed that a variety of tasks, communication, manager feedback, and stress management training were more highly valued by health workers with other levels of education and that work–life balance was more appreciated by health workers who hold higher school degrees (Table 9).

*Occupational groups*. Six psychosocial risk determinants (work overload, overtime, tight deadlines, unclear role, being under-skilled, and responsibility) and all organisational intervention objects (except stress management training) had mean rank scores differing statistically across occupational groups. Doctors reported the most negative perception of workload, overtime, and tight deadlines; nurses notified the most negative perception of being under-skilled; other health workers declared the most negative perception of unclear role; and heads of units marked the most negative perception of responsibility (Table 10). The highest scores almost for all organisational intervention objects was given by heads of units, and that only for variety of tasks was given by other health workers (Table 11).

## 4. Discussion

The study aimed to explore whether psychosocial risk determinants and organizational intervention objects differ across employees’ groups based on sociodemographic factors in a public primary healthcare institution. The findings of the study confirmed that different sociodemographic groups of health workers emphasized different psychosocial risk determinants and organisational intervention objects.

In fact, there was no difference between gender groups. Only one organisational intervention object, work–life balance, was more relevant to women than to men. This may be due to gender imbalances in the institution—as is the case in all Lithuanian primary healthcare institutions. However, previous research findings provide contradictory information on gender differences in wellbeing at work [21]. The study asserted that only one psychosocial risk determinant—excessive work pace, which has led to negative impact—increased consistently with age, contrary to previous research [22]. Regarding organizational interventions objects and age groups, the youngest group of health workers (≤30) emphasized more justice of reward, while the oldest group of health workers (>50) valued more matching to the job demands. A possible explanation for this might be that age might affect several components of the stress process at work; as these effects are partly conflicting, they might nullify each other in the overall relation between age and stress [23]. Only one psychosocial risk determinant and three organisational intervention objects were statistically significantly different among job seniority groups. Once more, excessive work pace had the greatest negative impact on health workers with the highest seniority (>10). Justice of reward, variety of tasks, and matching to the job demands were the most relevant to health workers who worked three to five years in the institution. These findings are original, but it can be due to specific standards and regulations in the Lithuanian public primary healthcare sector [24]. Compared with other groups, educational and professional groups significantly differed in their approach to psychosocial risk determinants and organizational intervention objects. Health workers who hold university degrees emphasized overtime, while health workers who hold higher school degrees stressed being under-skilled and health workers with other levels of education pointed out unclear role and conflicting roles. This is in contrast to Marinaccio’ findings [25] that showed that workers with highest level of education perceived more role ambiguity and had skills that exceeds their job requirements. Interpretation may be that these results were influenced by specificity of work in the public healthcare institution. Regarding organisational intervention objects, work–life balance was the most relevant to health workers who hold higher school degrees. Variety of tasks, communication, manager feedback, and stress management training were the most relevant to health workers with other levels of education, confirming that “having little chance of formal promotion prospect for professional acknowledgement and respect lie in building informal competence and local reputation” [25]. Furthermore, more than half of the psychosocial risk determinants significantly differed due to occupational groups. Responsibility for decision making and actions was the most psychosocial risk determinant per the perception of heads of units. Doctors stressed workload, overtime, and tight deadlines, while nurses emphasized being under-skilled and other health workers pointed out unclear roles. This may be considered as specificity of the public healthcare institution. Heads’ of units and doctors’ tasks may fit their knowledge, skills, and abilities needed to perform the job; nurses’ and others health workers’ tasks may go beyond the job descriptions. These results are in line with previous studies suggesting that nurses have a coordinating role, the responsibility for shuttling between professional, organisational, and relational tasks [26]. Surprisingly, all organisational intervention objects were the most relevant to the heads of units, except that variety of tasks was the most relevant to other health workers, while doctors perceived almost all organisational intervention objects (social support, organisational support, participation in decision making, communication, justice of reward, and manager feedback) as irrelevant. In line with the literature, the findings confirm that public sector doctors’ work is busier and more stressful than other health workers’ work, and this may lead to psychological problems and burnout [21,27,28].

In summary, the results are in accordance with the studies indicating that occupational groups are the key factor that should be considered when managing psychosocial risks at organisation [12,29,30,31]. Other sociodemographic groups such as gender, age, education, and job seniority should be considered, with caution as an additional information because studies were very controversial on how and whom they influence [22].

Nevertheless, this study also has limitations. First, the study is a cross-sectional study and cannot make conclusions regarding causality. Second, the findings of this study are restricted to the public healthcare institution. Third, it did not include individual intervention objects that focus on helping individual employees to develop skills to manage, cope with, and reduce stress at work, whereas organisation-level interventions address the health and wellbeing of relatively large groups of workers in a uniform way [32]. Fourth, the small number of male workers in the sample may affect other subgroups of health workers (e.g., heads of units or some categories of job seniority), but it is the case for many Lithuanian primary healthcare institutions. Notwithstanding its limitations, this study provides some support for an integrated approach to the consideration of target groups in order to diagnose psychosocial risk determinants and to tailor organisational interventions to their specific needs at the institution. Healthcare institutions are likely to comprise competing and overlapping professional groups, and a key challenge is to consider carefully the impact of change on specific groups (e.g. doctors, nurses, and other health professionals and managers) and to design appropriate policies [28,33].

## 5. Conclusions

The findings suggest that systematic assessment of risk groups on the basis of sociodemographic factors, especially occupational status, could facilitate psychosocial risk management in the public primary healthcare institution. It should be noted that the results of this study were based on a specific sample and that the method that was used to make internal comparisons of attitudes of different sociodemographic groups towards the psychosocial risk management inherently challenges generalizability and replicability of the results. Nevertheless, the findings might be generalized to countries with tax-financed universal healthcare system, considering the sociocultural context.

The study has proposed a way of diagnosing psychosocial risks and of tailoring interventions to all health workers of the public primary healthcare institution by using a simple and robust tool. For future research, it would be useful to examine attitudes of different sociodemographic groups towards the psychosocial risks and organizational intervention objects in a private primary healthcare institution. In addition, the study and the findings can be a basis for developing stress management programs in public healthcare institutions and an incentive for new theories of qualitative studies.

## Figures and Tables

**Table 1:** Groups on the basis of sociodemographic factors.

Groups		*N*	%
Gender	Male	24	5.1
	Female	443	94.9
	Total	467	100
Age	≤30	48	10.5
	31–40	68	14.8
	41–50	123	26.8
	>50	220	47.9
	Total	459	100
Education	University degree	247	52.9
	Higher school/college degree	180	38.5
	Other	40	8.6
	Total	467	100
Occupational status/groups	Heads of units	29	6.2
	Doctors	132	28.3
	Nurses	205	43.9
	Other health workers	101	21.6
	Total	467	100
Job seniority	≤3	49	10.7
	3.01–5	24	5.2
	5.01–10	37	8.0
	>10	350	76.1
	Total	460	100

*N*—sample sizes.

**Table 2:** Psychosocial risk determinants and gender results of the Mann–Whitney U-test.

Variables	Male		Female		*U*	*p*
	Mean Rank	*N*	Mean Rank	*N*		
Hazardous Working Conditions	259.38	24	232.63	443	4707.00	0.33
Work overload	245.38	24	233.38	443	5043.00	0.66
Excessive work pace	228.96	24	234.27	443	5195.00	0.84
Overtime	222.73	24	234.61	443	5045.50	0.66
Tight deadlines	250.25	24	233.12	443	4926.00	0.53
Unclear role	227.79	24	234.34	443	5167.00	0.81
Conflicting roles	260.98	24	232.54	443	4668.50	0.30
Being under-skilled for a job	210.65	24	235.27	443	4755.50	0.37
Responsibility for decision making and actions	250.25	24	233.12	443	4926.00	0.52
Lack of control over work pace	211.17	24	235.24	443	4768.00	0.38
Lack of control over work method	255.50	24	232.84	443	4800.00	0.40
Harassment	240.42	24	233.65	443	5162.00	0.79
Conflicts	253.29	24	232.95	443	4853.00	0.46
Tension	241.08	24	233.62	443	5146.00	0.78

Sample sizes—*N*, *U* values—the Mann–Whitney U-test, *χ*^2^ values, with k − 1 degrees of freedom - the Kruskall–Wallis test, significance levels—*p*.

**Table 3:** Organizational intervention objects and gender results of the Mann–Whitney U-test.

Variables	Male		Female		*U*	*p*
	Mean Rank	*N*	Mean Rank	*N*		
Work–Life Balance	170.38	24	237.45	443	3789.00	0.01
Skills/abilities matching to the job demands	204.90	24	235.58	443	4617.50	0.25
Variety of tasks	247.58	24	233.26	443	4990.00	0.60
Social support	215.81	24	234.99	443	4879.50	0.50
Organizational support	208.90	24	235.36	443	4713.50	0.35
Participation in decision making	198.54	24	235.92	443	4465.00	0.18
Communication	198.02	24	235.95	443	4452.50	0.18
Justice of reward	202.00	24	235.73	443	4548.00	0.23
Manager feedback	230.58	24	234.19	443	5234.00	0.90
Stress management training	230.63	24	234.18	443	5235.00	0.90

**Table 4:** Psychosocial risk determinants and age group results of the Kruskall–Wallis test.

Variables	≤30		]30–40]		]40–50]		>50		*X^2^*(3)	*p*
	Mean Rank	*N*	Mean Rank	*N*	Mean Rank	*N*	Mean Rank	*N*		
Hazardous Working Conditions	229.53	48	226.44	68	225.26	123	233.85	220	0.41	0.94
Work overload	212.23	48	227.40	68	218.34	123	241.2	220	3.58	0.31
Excessive work pace	177.81	48	207.74	68	227.13	123	249.87	220	17.36	<0.01
Overtime	199.69	48	229.10	68	233.84	123	234.75	220	3.21	0.36
Tight deadlines	209.72	48	243.88	68	242.37	123	223.22	220	3.80	0.28
Unclear role	198.13	48	217.11	68	224.30	123	244.13	220	6.63	0.08
Conflicting roles	208.38	48	208.74	68	254.01	123	227.87	220	7.60	0.05
Being under-skilled for a job	190.92	48	216.49	68	240.98	123	236.56	220	6.72	0.08
Responsibility for decision making and actions	253.69	48	236.07	68	220.58	123	228.22	220	2.69	0.44
Lack of control over work pace	277.73	48	232.26	68	222.39	123	223.14	220	7.63	0.05
Lack of control over work method	247.51	48	259.38	68	226.01	123	219.33	220	6.27	0.10
Harassment	231.72	48	233.09	68	246.96	123	219.19	220	4.30	0.23
Conflicts	229.10	48	253.68	68	224.42	123	226.00	220	2.76	0.43
Tension	228.82	48	246.32	68	224.31	123	228.39	220	1.40	0.71

**Table 5:** Organizational intervention objects and age group results of the Kruskall–Wallis test.

Variables	≤30		]30–40]		]40–50]		>50		*X^2^*(3)	*p*
	Mean Rank	*N*	Mean Rank	*N*	Mean Rank	*N*	Mean Rank	*N*		
Work–Life Balance	252.97	48	212.99	68	237.13	123	226.26	220	3.33	0.34
Skills/abilities matching to the job demands	202.26	48	189.69	68	232.65	123	247.03	220	13.41	<0.01
Variety of tasks	232.03	48	222.53	68	231.51	123	231.02	220	0.27	0.96
Social support	222.32	48	209.81	68	231.98	123	236.81	220	2.36	0.51
Organizational support	243.05	48	223.24	68	234.60	123	226.67	220	0.94	0.81
Participation in decision making	226.19	48	210.75	68	230.41	123	236.55	220	2.04	0.56
Communication	230.20	48	201.90	68	225.49	123	241.16	220	4.80	0.19
Justice of reward	278.61	48	247.19	68	235.06	123	211.25	220	12.44	<0.01
Manager feedback	223.95	48	228.51	68	230.33	123	231.60	220	0.14	0.99
Stress management training	191.66	48	218.25	68	238.30	123	237.36	220	6.00	0.11

**Table 6:** Psychosocial risk determinants and job seniority (working years) group results of the Kruskall–Wallis test.

Variables	≤3		]3–5]		]5–10]		>10		*X^2^*(3)	*p*
	Mean Rank	*N*	Mean Rank	*N*	Mean Rank	*N*	Mean Rank	*N*		
Hazardous Working Conditions	209.59	49	262.04	24	225.24	37	231.82	350	2.78	0.43
Work overload	221.77	49	206.17	24	215.36	37	234.99	350	1.99	0.57
Excessive work pace	181.03	49	238.38	24	173.26	37	242.94	350	20.36	<0.01
Overtime	200.22	49	264.67	24	232.39	37	232.20	350	4.67	0.20
Tight deadlines	223.78	49	248.33	24	206.72	37	232.73	350	2.00	0.57
Unclear role	214.83	49	189.77	24	210.69	37	237.58	350	5.14	0.16
Conflicting roles	201.95	49	253.58	24	213.88	37	234.67	350	4.17	0.24
Being under-skilled for a job	201.15	49	265.5	24	207.45	37	234.65	350	5.92	0.12
Responsibility for decision making and actions	245.51	49	238.48	24	227.64	37	228.15	350	0.97	0.81
Lack of control over work pace	266.15	49	185.44	24	206.46	37	231.14	350	7.92	0.05
Lack of control over work method	235.12	49	244.90	24	215.16	37	230.49	350	0.92	0.82
Harassment	230.80	49	236.67	24	255.22	37	227.42	350	1.86	0.60
Conflicts	215.26	49	272.52	24	251.55	37	227.53	350	4.42	0.22
Tension	229.86	49	240.65	24	228.07	37	230.15	350	0.17	0.98

**Table 7:** Organizational intervention objects and job seniority (working years) group results of the Kruskall–Wallis test.

Variables	≤3		]3–5]		]5–10]		>10		*X^2^*(3)	*p*
	Mean Rank	*N*	Mean Rank	*N*	Mean Rank	*N*	Mean Rank	*N*		
Work–Life Balance	238.47	49	259.69	24	212.14	37	229.32	350	2.23	0.53
Skills/abilities matching to the job demands	181.48	49	255.65	24	194.91	37	239.40	350	13.07	<0.01
Variety of tasks	220.63	49	323.31	24	228.92	37	225.68	350	12.98	<0.01
Social support	227.55	49	255.31	24	219.93	37	230.33	350	1.10	0.78
Organizational support	241.46	49	257.73	24	241.74	37	225.91	350	2.04	0.56
Participation in decision making	215.94	49	269.9	24	217.54	37	231.21	350	3.10	0.38
Communication	227.79	49	240.60	24	201.36	37	233.27	350	2.11	0.55
Justice of reward	288.12	49	290.73	24	268.65	37	214.27	350	22.91	<0.01
Manager feedback	212.77	49	280.75	24	230.74	37	229.51	350	4.34	0.23
Stress management training	191.08	49	241.29	24	230.18	37	235.31	350	5.18	0.16

**Table 8:** Psychosocial risk determinants and education group results of the Kruskall–Wallis test.

Variables	University		Higher School		Other		*X^2^*(2)	*p*
	Mean Rank	*N*	Mean Rank	*N*	Mean Rank	*N*		
Hazardous Working Conditions	229.97	247	238.26	180	239.71	40	0.49	0.78
Work overload	235.40	247	235.93	180	216.69	40	0.76	0.68
Excessive work pace	222.64	247	251.31	180	226.26	40	5.88	0.05
Overtime	249.66	247	215.19	180	221.95	40	7.93	0.02
Tight deadlines	234.83	247	235.31	180	223.00	40	0.32	0.85
Unclear role	207.93	247	262.09	180	268.59	40	21.26	<0.01
Conflicting roles	220.63	247	241.75	180	281.68	40	8.56	0.01
Being under-skilled for a job	208.45	247	263.37	180	259.63	40	20.22	<0.01
Responsibility for decision making and actions	242.40	247	228.50	180	206.85	40	3.32	0.19
Lack of control over work pace	244.37	247	221.86	180	224.63	40	3.28	0.19
Lack of control over work method	242.11	247	225.11	180	223.95	40	2.08	0.35
Harassment	226.46	247	237.20	180	266.13	40	3.84	0.15
Conflicts	230.51	247	237.87	180	238.10	40	0.37	0.83
Tension	233.95	247	233.01	180	238.78	40	0.06	0.97

**Table 9:** Organizational intervention objects and education group results of the Kruskall–Wallis test.

Variables	University		Higher School		Other		*X^2^*(2)	*p*
	Mean Rank	*N*	Mean Rank	*N*	Mean Rank	*N*		
Work–Life Balance	223.88	247	253.88	180	207.00	40	7.44	0.02
Skills/abilities matching to the job demands	229.74	247	239.09	180	237.40	40	0.59	0.74
Variety of tasks	208.24	247	257.81	180	285.91	40	21.42	<0.01
Social support	226.35	247	238.62	180	260.44	40	2.56	0.28
Organizational support	229.88	247	235.19	180	254.09	40	1.14	0.56
Participation in decision making	224.28	247	239.38	180	269.85	40	4.45	0.11
Communication	217.23	247	247.41	180	277.24	40	9.80	<0.01
Justice of reward	233.40	247	229.30	180	258.84	40	1.61	0.45
Manager feedback	227.04	247	228.60	180	301.30	40	10.95	<0.01
Stress management training	211.19	247	254.09	180	284.45	40	17.49	<0.01

**Table 10:** Psychosocial risk determinants and occupational group results of the Kruskall–Wallis test.

Variables	Heads of the Units		Doctors		Nurses		Other		*X^2^*(3)	*p*
	Mean Rank	*N*	Mean Rank	*N*	Mean Rank	*N*	Mean Rank	*N*		
Hazardous Working Conditions	169.83	29	235.16	132	236.41	205	246.01	101	7.79	0.05
Work overload	187.41	29	263.63	132	232.77	205	211.15	101	13.41	<0.01
Excessive work pace	230.62	29	242.72	132	232.08	205	227.47	101	1.03	0.79
Overtime	245.28	29	263.42	132	229.85	205	200.73	101	14.23	<0.01
Tight deadlines	212.28	29	257.47	132	233.84	205	209.89	101	8.64	0.03
Unclear role	152.5	29	226.68	132	239.14	205	256.53	101	15.24	<0.01
Conflicting roles	219.72	29	224.91	132	228.67	205	260.80	101	5.58	0.13
Being under-skilled for a job	193.55	29	212.52	132	251.81	205	237.53	101	10.30	0.02
Responsibility for decision making and actions	282.62	29	252.87	132	230.21	205	203.07	101	13.66	<0.01
Lack of control over work pace	211.07	29	243.61	132	241.97	205	211.85	101	5.22	0.16
Lack of control over work method	252.97	29	234.89	132	240.40	205	214.41	101	3.48	0.32
Harassment	200.97	29	231.59	132	238.73	205	237.02	101	2.55	0.47
Conflicts	222.72	29	220.33	132	249.84	205	222.95	101	5.39	0.14
Tension	207.55	29	222.98	132	246.24	205	231.16	101	4.03	0.26

**Table 11:** 0rganizational intervention objects and occupational group results of the Kruskall–Wallis test.

Variables	Heads of the Units		Doctors		Nurses		Other		*X^2^*(3)	*p*
	Mean Rank	*N*	Mean Rank	*N*	Mean Rank	*N*	Mean Rank	*N*		
Work–Life Balance	282.10	29	202.67	132	244.51	205	239.81	101	13.19	<0.01
Skills/abilities matching to the job demands	295.91	29	240.48	132	238.66	205	198.30	101	15.29	<0.01
Variety of tasks	152.57	29	203.43	132	228.24	205	309.02	101	51.06	<0.01
Social support	295.64	29	213.57	132	236.10	205	238.73	101	9.33	0.02
Organizational support	332.00	29	218.53	132	235.45	205	223.12	101	17.88	<0.01
Participation in decision making	295.64	29	217.84	132	235.33	205	234.72	101	8.08	0.04
Communication	270.62	29	204.50	132	241.47	205	246.87	101	10.10	0.02
Justice of reward	292.10	29	207.33	132	230.25	205	259.78	101	14.70	<0.01
Manager feedback	308.00	29	215.63	132	223.57	205	257.94	101	15.65	<0.01
Stress management training	235.90	29	214.92	132	244.20	205	237.68	101	4,09	0.25
